# Interrogating conserved transcriptomic signatures of cognitive resilience in the frontal cortex

**DOI:** 10.1002/alz70855_096281

**Published:** 2025-12-23

**Authors:** Lauren A Fish, Saranya Canchi, Maria Telpoukhovskaia, Tain Luquez, Jonathan Algoo, Niran Hadad, Brianna Gurdon, Miko Dai, Andrew R Ouellette, Sarah M Neuner, Amy R Dunn, Jon A. L. Willcox, Yiyang Wu, Logan Dumitrescu, Orhan Bellur, Jigang Zhang, Kristen MS O'Connell, Eric B. Dammer, Nicholas T Seyfried, Sukalp Muzumdar, Jesse Gillis, Paul Robson, Matthias Arnold, Timothy J. Hohman, Vivek M. Philip, Vilas Menon, Catherine C. Kaczorowski

**Affiliations:** ^1^ University of Michigan, Ann Arbor, MI, USA; ^2^ The Jackson Laboratory, Bar Harbor, ME, USA; ^3^ Columbia University Irving Medical Center, New York, NY, USA; ^4^ Columbia University, New York, NY, USA; ^5^ The University of Maine, Orono, ME, USA; ^6^ University of Maine Graduate School of Biomedical Science and Engineering, Orono, ME, USA; ^7^ Icahn School of Medicine at Mount Sinai, New York, NY, USA; ^8^ Vanderbilt Memory and Alzheimer's Center, Institute for Medicine and Public Health, Vanderbilt University Medical Center, Nashville, TN, USA; ^9^ Vanderbilt Memory and Alzheimer's Center, Vanderbilt University Medical Center, Nashville, TN, USA; ^10^ Institute of Computational Biology, Helmholtz Zentrum München, German Research Center for Environmental Health, Neuherberg, Germany; ^11^ Emory University School of Medicine, Atlanta, GA, USA; ^12^ Cold Spring Harbor, Cold Spring Harbor, NY, USA; ^13^ University of Toronto, Toronto, ON, Canada; ^14^ University of Connecticut, Farmington, CT, USA; ^15^ The Jackson Laboratory for Genomic Medicine, Farmington, CT, USA; ^16^ Helmholtz Zentrum Muenchen, Oberschleißheim, Germany; ^17^ Duke University, Durham, NC, USA; ^18^ Vanderbilt Memory & Alzheimer's Center, Vanderbilt University Medical Center, Nashville, TN, USA; ^19^ Center for Translational & Computational Neuroimmunology, Columbia University Irving Medical Center, New York, NY, USA

## Abstract

**Background:**

Development of effective drugs for Alzheimer's Disease (AD) is challenging, likely due in part to poor recapitulation of AD in animal models. We developed the genetically diverse AD‐BXD mouse model, a panel of reproducible strains that exhibit variation in age at onset and extent of cognitive decline observed in human AD. Using multi‐modal data (genomic, protein, behavior) from this model, we have uncovered new genes and pathways mediating cognitive resilience to AD. For example, we recently reported a resilience gene expression signature in excitatory intratelencephalic neurons (Telpoukhovskaia et al., 2023, *bioRxiv*).

**Method:**

To uncover novel, conserved gene expression signatures and neuronal subtypes associated with AD resilience, we integrated frontal cortex AD‐BXD single‐nuclei transcriptomic data (*N* = 56) with a larger human dataset (*N* = 465, ROSMAP, Green et. al, 2024, *Nature)* to generate translationally relevant mouse cell annotations. After integration and annotation of the mouse data with the human cell taxonomy, we conducted differential gene expression (DE) analysis on mouse excitatory and inhibitory neuronal subclasses using animals’ performance on a fear conditioning task as a continuous cognitive resilience covariate (negative binomial mixed models accounting for subject‐ and cell‐level overdispersion with Nebula).

**Result:**

In benchmarking experiments, we confirmed that the continuous cognitive metric confers higher statistical power than a conventional categorical resilience covariate while retaining most DE genes found using the categorical covariate. In the current analysis, we identified DE genes in excitatory and inhibitory neuronal subtypes, with the largest number of DE genes found in layer 2/3 excitatory neurons (markers LINC00507, GLRA3, RORB). Several of the DE genes from this cluster have already been studied in the context of neurodegeneration. SCG5, the only DE gene found in both an excitatory and an inhibitory neuronal cluster, was also recently shown to be associated with AD risk.

**Conclusion:**

We identified a transcriptomic signature associated with cognitive resilience in a genetically diverse AD mouse model. Ongoing work aims to interrogate resilience gene expression signatures and cell types that are conserved across mice and humans. Downstream analyses will include unbiased drug target nomination and drug repositioning analyses to prioritize promising genes, pathways, and drugs for preclinical validation.

